# Effects of 12 Weeks of Combined Exercise Training in Normobaric Hypoxia on Arterial Stiffness, Inflammatory Biomarkers, and Red Blood Cell Hemorheological Function in Obese Older Women

**DOI:** 10.3390/healthcare12181887

**Published:** 2024-09-20

**Authors:** Wonil Park, Hun-Young Park, Sung-Woo Kim

**Affiliations:** 1 Department of Sports Science, Korea Institute of Sports Science, 424 Olympic-ro, Songpa-gu, Seoul 05540, Republic of Korea; wonilpark01@konkuk.ac.kr; 2 Department of Sports Medicine and Science, Graduate School, Konkuk University, 120 Neungdong-ro, Gwangjin-gu, Seoul 05029, Republic of Korea; parkhy1980@konkuk.ac.kr; 3 Physical Activity and Performance Institute, Konkuk University, 120 Neungdong-ro, Gwangjin-gu, Seoul 05029, Republic of Korea

**Keywords:** normobaric hypoxia, combined exercise, arterial stiffness, inflammatory biomarkers, hemorheological function

## Abstract

Background/Objectives: The present study examined the effect of 12-week combined exercise training in normobaric hypoxia on arterial stiffness, inflammatory biomarkers, and red blood cell (RBC) hemorheological function in 24 obese older women (mean age: 67.96 ± 0.96 years). Methods: Subjects were randomly divided into two groups (normoxia (NMX; n = 12) and hypoxia (HPX; n = 12)). Both groups performed aerobic and resistance exercise training programs three times per week for 12 weeks, and the HPX group performed exercise programs in hypoxic environment chambers during the intervention period. Body composition was estimated using bioelectrical impedance analysis equipment. Arterial stiffness was measured using an automatic waveform analyzer. Biomarkers of inflammation and oxygen transport (tumor necrosis factor alpha, interleukin 6 (IL-6), erythropoietin (EPO), and vascular endothelial growth factor (VEGF)), and RBC hemorheological parameters (RBC deformability and aggregation) were analyzed. Results: All variables showed significantly more beneficial changes in the HPX group than in the NMX group during the intervention. The combined exercise training in normobaric hypoxia significantly reduced blood pressure (systolic blood pressure: *p* < 0.001, diastolic blood pressure: *p* < 0.001, mean arterial pressure: *p* < 0.001, pulse pressure: *p* < 0.05) and brachial–ankle pulse wave velocity (*p* < 0.001). IL-6 was significantly lower in the HPX group than in the NMX group post-test (*p* < 0.001). Also, EPO (*p* < 0.01) and VEGF (*p* < 0.01) were significantly higher in the HPX group than in the NMX group post-test. Both groups showed significantly improved RBC deformability (RBC EI_3Pa) (*p* < 0.001) and aggregation (RBC AI_3Pa) (*p* < 0.001). Conclusions: The present study suggests that combined exercise training in normobaric hypoxia can improve inflammatory biomarkers and RBC hemorheological parameters in obese older women and may help prevent cardiovascular diseases.

## 1. Introduction

In recent years, the global prevalence of obesity among older women has risen significantly, posing a substantial public health challenge [1]. In the face of the worldwide obesity epidemic and its associated cardiovascular complications, there is a growing interest in innovative therapeutic strategies to mitigate the adverse health effects of excess adiposity, particularly in aging populations [2,3,4,5]. As a multifaceted condition, obesity is closely linked to increased arterial stiffness, elevated cardiometabolic risk factors (CMRFs), and alterations in red blood cell (RBC) hemorheological function, contributing to a higher risk of cardiovascular events [5,6,7,8,9]. Obesity is intricately linked to an increased risk of cardiovascular diseases (CVDs) and metabolic complications, necessitating targeted interventions to address these health concerns [10,11]. Exercise training has long been established as a cornerstone in managing obesity and related metabolic disorders. Recent research suggests that exercise, particularly in a normobaric hypoxic environment, is a promising strategy to enhance cardiovascular function and mitigate CMRFs [12,13,14,15]. A systematic review of a randomized controlled trial reported improvements in weight, body mass index (BMI), waist circumference, waist–hip ratio, fat mass, lean mass, and systolic blood pressure (SBP) in a hypoxic group compared with baseline [16]. Therefore, these results suggest that combining hypoxia and exercise may help improve CMRFs in obese individuals [16].

Normobaric hypoxia, characterized by reduced atmospheric pressure and oxygen availability, creates a unique physiological environment that challenges the cardiovascular and respiratory systems [12,14,15,17,18]. Previous studies have suggested that exposure to normobaric hypoxia may induce adaptive responses, such as increased RBC mass, improved oxygen transport capacity, and enhanced cardiovascular function [17,18,19,20,21]. While exercise training alone has been proven effective in addressing specific aspects of obesity-related health and aging issues, the synergistic effects of normobaric hypoxia and exercise on arterial function, metabolic markers, and RBC function in the context of obesity among older women remain relatively unexplored.

One proposed mechanism in the previous study suggests that exercise can modulate arterial stiffness and inflammation in obesity through various pathways [22,23]. Regular physical activity has been shown to improve endothelial function, reduce oxidative stress, and decrease inflammation markers such as C-reactive protein and interleukin 6 (IL-6) [24,25]. Additionally, exercise promotes the release of vasodilatory substances such as nitric oxide, which helps to maintain vascular health [26]. Hypoxic stress, a unique condition induced by exercises performed under reduced oxygen levels or at high altitudes, may offer additional benefits by enhancing some of these mechanisms [27,28]. Hypoxia can stimulate the release of vascular endothelial growth factor (VEGF) and erythropoietin (EPO), which promote angiogenesis and erythropoiesis, respectively [29,30]. These responses could improve vascular function and reduce inflammation, providing unique benefits beyond exercise alone. Overall, exercise and hypoxic stress may synergistically modulate arterial stiffness and inflammation in obesity by improving vascular function, reducing oxidative stress, and promoting anti-inflammatory responses.

Arterial stiffness is a well-established marker of cardiovascular health, reflecting changes in the elasticity of arterial walls and providing insights into vascular aging and overall cardiovascular risk [31]. Arterial stiffness is a crucial determinant of cardiovascular health and is often elevated in obesity [22]. Additionally, obese individuals face elevated risks of metabolic dysfunction, insulin resistance, and related cardiometabolic complications [22,31]. Exercise training has been established as a potent modulator of arterial stiffness, and the addition of hypoxic stress may further potentiate these effects [5,32]. Understanding the impact of hypoxic exercise on arterial stiffness in obese older women holds significant clinical relevance, as it may provide insights into optimizing interventions for this vulnerable population. Concurrently, alterations in CMRFs, such as glucose metabolism, lipid profiles, and inflammatory markers, play pivotal roles in the pathogenesis of CVD associated with obesity [5,8,32]. The intricate interplay between exercise, hypoxia, and metabolic health in the context of obesity in older women remains a relatively underexplored area, making this research particularly timely and valuable. Understanding how exercise in normobaric hypoxia modulates CMRFs can contribute to the development of targeted interventions for managing obesity-related cardiovascular and metabolic complications in older women.

Furthermore, RBC hemorheological function, which encompasses RBC deformability and aggregation properties, contributes to the microcirculatory dynamics and may influence overall cardiovascular function [33,34]. RBC deformability and rheological properties are pivotal in microcirculatory function and tissue oxygenation [33,34]. These parameters play a crucial role in oxygen delivery to tissues and overall cardiovascular function, warranting a comprehensive exploration of their response to hypoxic exercise in the context of obesity. Evaluating the influence of hypoxic exercise on RBC function contributes to our understanding of the integrated physiological adaptations induced by this unique training paradigm [34,35].

In summary, this research advances our understanding of the synergistic effects of combined exercise training and normobaric hypoxia on arterial stiffness, inflammatory biomarkers, and RBC hemorheological function in obese older women. The findings from this research hold the potential to inform novel and tailored exercise strategies that may optimize cardiovascular health outcomes in the ever-growing demographic of older individuals grappling with obesity-related complications. Therefore, this study hypothesizes that 12 weeks of combined exercise in normobaric hypoxia will affect arterial stiffness, inflammatory biomarkers, and RBC hemorheological function in obese older women.

## 2. Materials and Methods

### 2.1. Participants

Using G*Power 3.1.9.2 (Franz Faul, University of Kiel, Kiel, Germany), a power analysis was conducted with a power of 0.80, an effect size of 0.3, and a significance level of 0.05 [8]. This analysis determined that a sample size of 24 participants was optimal. Initially, 30 obese women aged 67–70 years (age: 67.96 ± 0.96 years) were enrolled in the study. The inclusion criteria required participants to have a BMI > 25 kg·m^−2^ (27.09 ± 0.96 kg·m^−2^), percentage body fat > 30% (32.79 ± 2.00%), and low levels of physical activity (no exercise in the last six months and fewer than 150 min of physical activity per week). Exclusion criteria included the presence of uncontrolled chronic diseases, a history of acute myocardial infarction, recent joint replacement or lower limb fracture within the past six months, and severe cognitive impairment. Participants were randomly assigned to either a normoxia (NMX) group or hypoxia (HPX) group. A computer-generated random order was used, and assignments were concealed from participants using sealed opaque envelopes. The physical characteristics of the participants are summarized in Table 1. Prior to the study, all participants were informed about the objectives, procedures, potential benefits, and possible risks involved. Written informed consent was obtained from each participant. The study was approved by the Institutional Review Board of Kyunghee University (KHSIRB-16-016) and was conducted in accordance with the principles of the Declaration of Helsinki. Additionally, the study was registered with the Clinical Research Information Service (http://cris.nih.go.kr) under the World Health Organization International Clinical Trials Registry Platform, with the registration number KCT0003341.

### 2.2. Study Design

The experimental design involved 1-day pre-testing, a 12-week session of the intervention period, and 1-day post-testing. The 12-week training session was performed in two-day intervals between pre- and post-testing to avoid intervention effects. Participants were prohibited from taking drugs and drinking coffee, which could affect measurement variables, until the test was completed. During the intervention period, a questionnaire was used to check potential confounding factors such as dietary intake, physical activity outside the intervention, and drug use. In addition, participants were immediately stopped when they had health problems pre-, post-test, or during exercise intervention. On the pre- and post-testing days, all participants underwent fasting for more than 10 h, and after stabilization, venous blood was collected between 8:00 and 9:00 a.m. Then, after a 20 min break, body composition, BP, and arterial stiffness parameters were measured in that order in the morning.

All participants performed the following three combined exercise interventions for 120 min. Aerobic exercises were conducted after elastic-band resistance exercises in the order of the training program [9]. The HPX group experiments were performed in a 6.5 × 7.5 × 3 m (width × length × height) environmental chamber (Submersible Systems, Huntington Beach, CA, USA) [17]. Various hypoxic conditions were simulated by introducing nitrogen into the environmental chamber using a nitrogen generator (Separation & Filter Energy Technology Cooperation, Siheung, Republic of Korea) with the capacity to simulate hypoxic conditions for altitudes of up to 14.5% O_2_, equivalent to an altitude of 3000 m. All exercise interventions were performed at a constant temperature and humidity (23 ± 1 °C, 50 ± 5%) for 12 weeks three times a week. Resistance exercise-priority order appears to be more effective in improving aerobic capacity in older adults and women and affects metabolism in favor of subsequent aerobic sessions [36].

All older obese women participated in resistance training sessions that included exercises such as squats, incline chest presses, seated rows, push presses, split squats, and pull-aparts. Each participant completed three sets of 10–15 repetitions, with the exercise intensity rated between 7 and 8 on the OMNI Resistance Exercise Scale of Perceived Exertion (OMNI-RES AM), where 0 represents “extremely easy” and 10 represents “extremely hard.” This intensity range corresponds to approximately 70–80% of one repetition maximum (1 RM). Participants rested for 90 s between sets, and the resistance training sessions lasted about 35 min. For aerobic exercise, the participants’ maximum heart rate (HR_max_) was calculated using the Tanaka formula: HR_max_ = 208 − (0.7 × age). They then performed 60 min of aerobic exercise at an intensity corresponding to 60–70% of their HRmax. The aerobic exercise session was divided into two 30 min segments, starting with treadmill exercise followed by cycling. Participants were instructed to monitor their target heart rate using a heart rate monitor (M400, Polar, Helsinki, Finland) worn on the chest during the aerobic exercise session. Aerobic exercises were performed after the resistance training session, with a 15 min rest period in between. Throughout the 12-week supervised exercise intervention, participants received specialized instruction and coaching from an experienced trainer, ensuring that no adverse events related to the exercise regimen occurred. Table 2 provides detailed information about the combined exercise intervention program.

### 2.3. Body Composition

Body composition (height, weight, fat mass, fat-free mass (FFM), and percentage body fat) was measured after fasting for more than six hours using bioelectrical impedance analysis equipment (Inbody 770, Seoul, Republic of Korea).

### 2.4. Blood Pressure and Arterial Stiffness

Blood pressure (BP) and brachial–ankle pulse wave velocity (baPWV) were measured using an automatic waveform analyzer (VP-1000, Colin Co., Komaki, Japan) in the supine position after at least 10 min of steady-state resting. Brachial–ankle pulse and waves were recorded with sensor cuffs on both upper arms and ankles. SBP, mean arterial pressure (MAP), diastolic blood pressure (DBP), and pulse pressure (PP) were analyzed. Means of the data on both sides were used in our study.

### 2.5. Biomarkers of Inflammation and Oxygen Transport

Biomarkers related to inflammation and oxygen transport were analyzed by the Green Cross Medical Foundation, a certified organization affiliated with the Korean Society for Laboratory Medicine. The following blood variables were quantified: tumor necrosis factor alpha (TNF-α), IL-6, EPO, and VEGF. A 6 mL venous blood sample was collected in a serum-separating tube (SST). The sample was allowed to clot and was then centrifuged at 3500 rpm for 10 min to separate the serum. TNF-α and IL-6 concentrations were determined using an enzyme immunoassay system (Bio-Rad Laboratories, Hercules, CA, USA) with reagents from R&D Systems (Minneapolis, MN, USA) based on the ELISA method. R&D Systems’ TNF-α and IL-6 ELISA kits have around 0.5–5 pg·mL^−1^ sensitivities. EPO levels were measured with an Immulite 2000 XPI analyzer (Siemens, Eschborn, Germany) using the chemiluminescent immunoassay method. Chemiluminescent immunoassays, like the one used in the Immulite 2000 XPI analyzer, are often reported to have a sensitivity of 0.5 to 2.0 IU·mL^−1^. VEGF concentrations were assessed using the enzyme-linked immunosorbent assay (ELISA) method with Quantikine ELISA kits from R&D Systems (Minneapolis, MN, USA). The lower detection limit for VEGF assays using Quantikine ELISA kits is usually 1 to 5 pg·mL^−1^. All blood assays were performed in duplicate to ensure accuracy.

### 2.6. Red Blood Cell Hemorheological Function

To evaluate RBC hemorheological function, we measured RBC deformability and aggregation [8]. Following the guidelines set by Uyuklu et al. [37], RBC deformability and aggregation were analyzed at 25 °C and shear stress of 3 Pa within 4–6 h after blood collection. To comply with these recommendations, all samples were analyzed within 30 min of collection at room temperature (25 °C) using a Rheoscan-D (Rheo Meditech Inc., Seoul, Republic of Korea). For the RBC elongation index (EI) analysis, the blood sample was transferred to a 2 mL microfuge tube and diluted with 700 μL of 5.5% polyvinylpyrrolidone dissolved in 1 mmol phosphate-buffered saline contained in a K3EDTA tube (Greiner Bio-One, Chon Nuri, Thailand). Subsequently, 0.5 mL of this solution was analyzed using a D-test kit following the manufacturer’s instructions (Rheo Meditech Inc., Seoul, Republic of Korea). To ensure accuracy in RBC EI measurement, the Lineweaver–Burk plot model was employed [38]. For the RBC aggregation index (AI) analysis, 8 μL of the whole-blood sample was analyzed directly using an A-test kit according to the manufacturer’s instructions (Rheo Meditech Inc., Seoul, Republic of Korea).

### 2.7. Statistical Analysis

Statistical analyses were conducted using IBM SPSS Statistics version 28.0 (IBM Corp., Armonk, NY, USA). Descriptive statistics, including mean values and standard deviations, were calculated for all variables. The normality of the distribution for each dependent variable was assessed using the Kolmogorov–Smirnov test. To evaluate group-by-time interaction effects during the intervention, a two-way repeated-measure analysis of variance (ANOVA) was performed. If significant interactions or main effects were detected, independent *t*-tests and paired *t*-tests were used to analyze within-group and between-group differences, respectively [8]. Effect sizes were computed as partial eta-squared (η_p_^2^) values, with thresholds for small (≥0.01), medium (≥0.06), and large (≥0.14) effects [39]. Statistical significance was set at *p* < 0.05.

## 3. Results

### 3.1. Body Composition

Significant group-by-time interaction effects were observed for weight (*F* = 14.798, *p* < 0.001, η_p_^2^ = 0.402), BMI (*F* = 17.229, *p* < 0.001, η_p_^2^ = 0.439), FFM (*F* = 5.617, *p* < 0.05, η_p_^2^ = 0.203), and percentage body fat (*F* = 93.026, *p* < 0.001, η_p_^2^ = 0.809) (Table 3). Also, significant time effects were observed for weight (*F* = 86.760, *p* < 0.001, η_p_^2^ = 0.798), BMI (*F* = 103.405, *p* < 0.001, η_p_^2^ = 0.825), FFM (*F* = 9.783, *p* < 0.01, η_p_^2^ = 0.308), and percentage body fat (*F* = 431.735, *p* < 0.001, η_p_^2^ = 0.952). There was no statistically significant difference in the group effect.

The paired *t*-test results showed that variables with significant interaction effects had significantly beneficial changes in both the NMX (weight: −1.88 kg, *p* < 0.001; BMI: −0.71 kg·m^−2^, *p* < 0.001; percentage body fat: −1.55%, *p* < 0.001) and HPX groups (weight: −4.53 kg, *p* < 0.001; BMI: −1.68 kg·m^−2^, *p* < 0.001; FFM: 1.20 kg, *p* < 0.01; percentage body fat: −1.55%, *p* < 0.001). Moreover, significant post-test differences were observed in percentage body fat (*p* < 0.05) between the NMX and HPX groups.

### 3.2. Blood Pressure and Arterial Stiffness

**Figure 1:**
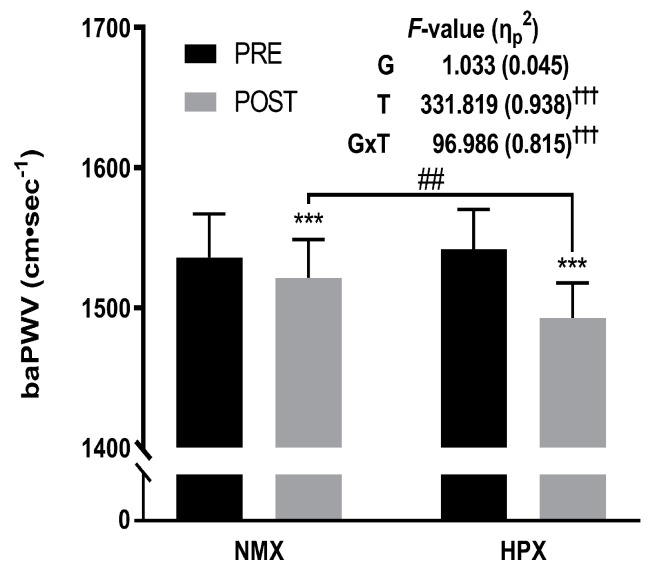
Arterial stiffness before and after the 12-week training program. For arterial stiffness, statistical analyses revealed a decrease between pre- and post-test parameters in both groups. NMX = normoxia, HPX = hypoxia. Significant interaction or main effect, ††† *p* < 0.001. Significant difference between pre- and post-tests, *** *p* < 0.001. Significant difference between NMX and HPX groups, ## *p* < 0.01.

The BP and arterial stiffness changes are presented in Table 4 and Figure 1. Significant group-by-time interaction effects were observed for SBP (*F* = 44.410, *p* < 0.001, η_p_^2^ = 0.669), DBP (*F* = 191.061, *p* < 0.001, η_p_^2^ = 0.897), MAP (*F* = 272.335, *p* < 0.001, η_p_^2^ = 0.925), PP (*F* = 6.821, *p* < 0.05, η_p_^2^ = 0.237), and baPWV (*F* = 96.986, *p* < 0.001, η_p_^2^ = 0.815). Also, significant time effects were observed for SBP (*F* = 323.418, *p* < 0.001, η_p_^2^ = 0.936), DBP (*F* = 223.983, *p* < 0.001, η_p_^2^ = 0.911), MAP (*F* = 598.183, *p* < 0.001, η_p_^2^ = 0.965), PP (*F* = 27.425, *p* < 0.001, η_p_^2^ = 0.555), and baPWV (*F* = 331.819, *p* < 0.001, η_p_^2^ = 0.938). The group effect was statistically significant only for MAP (*F* = 5.967, *p* < 0.05, η_p_^2^ = 0.213).

The paired *t*-test results showed that variables with significant interaction effects had significantly beneficial changes in both the NMX (SBP: −2.58 mmHg, *p* < 0.001; MAP: −0.99 mmHg, *p* < 0.001; PP: −2.39 mmHg, *p* < 0.001; baPWV: −14.63 cm·s^−1^, *p* < 0.001) and HPX groups (SBP: −5.63 mmHg, *p* < 0.001; DBP: −4.83 mmHg, *p* < 0.001; MAP: −5.09 mmHg, *p* < 0.001; baPWV: −49.05 cm·s^−1^, *p* < 0.001). Moreover, significant post-test differences were observed in SBP (*p* < 0.01), DBP (*p* < 0.01), MAP (*p* < 0.001), and baPWV (*p* < 0.01) between the NMX group and the HPX group.

### 3.3. Biomarkers of Inflammation and Oxygen Transport

**Figure 2:**
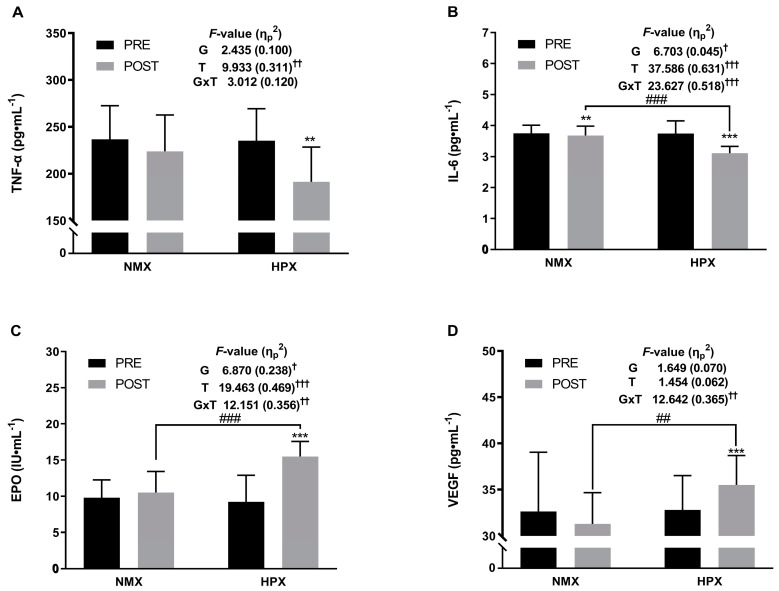
Biomarkers of inflammation and oxygen transport before and after the 12-week training program. (**A**) For TNF-α, statistical analyses revealed a decrease between pre- and post-test parameters in the HPX group. (**B**) For IL-6, statistical analyses revealed a decrease between pre- and post-test parameters in both groups. (**C**) For EPO, statistical analyses revealed an increase between pre- and post-test parameters in the HPX group. (**D**) For VEGF, statistical analyses revealed an increase between pre- and post-test parameters in the HPX group. NMX = normoxia, HPX = hypoxia, TNF-α = tumor necrosis factor alpha, IL-6 = interleukin 6, EPO = erythropoietin, VEGF = vascular endothelial growth factor. Significant interaction or main effect, † *p* < 0.05, †† *p* < 0.01, ††† *p* < 0.001. Significant difference between pre- and post-tests, ** *p* < 0.01, *** *p* < 0.001. Significant difference between NMX and HPX groups, ## *p* < 0.01, ### *p* < 0.001.

The changes in biomarkers of inflammation and oxygen transport are presented in Figure 2. Significant group-by-time interaction effects were observed for IL-6 (*F* = 23.627, *p* < 0.001, η_p_^2^ = 0.518), EPO (*F* = 12.151, *p* < 0.01, η_p_^2^ = 0.356), and VEGF (*F* = 12.642, *p* < 0.01, η_p_^2^ = 0.365). Also, significant time effects were observed for TNF-α (*F* = 9.933, *p* < 0.01, η_p_^2^ = 0.311), IL-6 (*F* = 37.586, *p* < 0.001, η_p_^2^ = 0.631), and EPO (*F* = 19.463, *p* < 0.001, η_p_^2^ = 0.469). The group effects were statistically significant for IL-6 (*F* = 6.703, *p* < 0.05, η_p_^2^ = 0.234) and EPO (*F* = 6.870, *p* < 0.05, η_p_^2^ = 0.238).

The paired *t*-test results showed that variables with significant interaction effects had significantly beneficial changes in both the NMX (IL-6: −0.07 pg·mL^−1^, *p* < 0.01) and HPX groups (TNF-α: −43.97 pg·mL^−1^, *p* < 0.01; IL-6: −0.63 pg·mL^−1^, *p* < 0.001; EPO: 6.28 IU·mL^−1^, *p* < 0.001; VEGF: 2.69 pg·mL^−1^, *p* < 0.001). Moreover, significant post-test differences were observed in IL-6 (*p* < 0.001), EPO (*p* < 0.001), and VEGF (*p* < 0.01) between the NMX group and the HPX group.

### 3.4. Red Blood Cell Hemorheological Function

**Figure 3:**
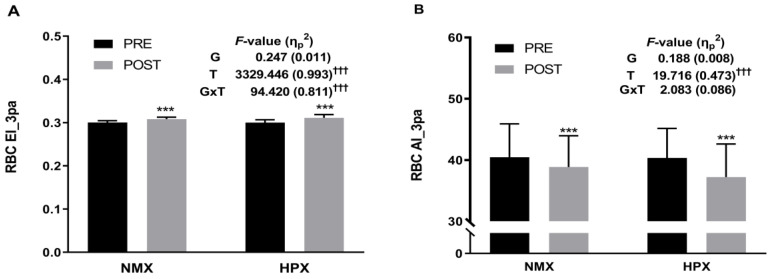
RBC hemorheological parameters before and after the 12-week training program. (**A**) For RBC deformability, statistical analyses revealed an increase between pre- and post-test parameters in both groups. (**B**) For RBC aggregation, statistical analyses revealed a decrease between pre- and post-test parameters in both groups. NMX = normoxia, HPX = hypoxia, RBC = red blood cell, EI = elongation index, AI = aggregation index. Significant interaction or main effect, ††† *p* < 0.001. Significant difference between pre- and post-tests, *** *p* < 0.001.

The changes in RBC hemorheological function are presented in Figure 3. Significant group-by-time interaction effects were observed for RBC EI (*F* = 94.420, *p* < 0.001, η_p_^2^ = 0.811). Also, significant time effects were observed for RBC EI (F = 3329.446, *p* < 0.001, η_p_^2^ = 0.993) and RBC AI (*F* = 19.716, *p* < 0.001, η_p_^2^ = 0.473). There was no statistically significant difference in the group effect.

The paired *t*-test results showed that variables with significant interaction effects had significantly beneficial changes in both the NMX (RBC EI: 0.01, *p* < 0.001; RBC AI: −1.58, *p* < 0.01) and HPX groups (RBC EI: 0.01, *p* < 0.001; RBC AI: −3.11, *p* < 0.01).

## 4. Discussion

The present study examined the effects of 12 weeks of combined exercise training in normobaric hypoxia on arterial stiffness, inflammatory biomarkers, and RBC hemorheological function in obese older women. Our main findings indicated significantly beneficial changes in body composition, arterial stiffness, inflammatory biomarkers, and RBC hemorheological function in the HPX group over the NMX group post-test. Thus, combined exercise training in normobaric hypoxia can prevent CMRFs in obese older women.

The detrimental changes in body composition of older women may encompass increased body fat, decreased FFM, reduced bone density, and alterations in distribution of fat, heightening susceptibility to metabolic disorders and compromising mobility and overall health [40,41]. The results of this study provide valuable insights into the effects of exercise under normoxic and hypoxic conditions on various body composition parameters, including weight, BMI, FFM, and percentage body fat. The findings revealed that both normoxic and hypoxic exercise interventions resulted in significant reductions in weight, BMI, and percentage body fat. However, the magnitude of these reductions was greater in the HPX group than in the NMX group. These results are consistent with previous studies that have demonstrated the potential benefits of hypoxic training for weight loss and body composition improvement due to increased energy expenditure and metabolic rate under hypoxic conditions [5,42,43]. The observed differences between the two exercise interventions suggest that exercising under hypoxic conditions may have augmented effects on weight loss and fat reduction compared to exercising under normoxic conditions [5,42,43]. Moreover, the increase in FFM in the HPX group suggests that exercising in hypoxic conditions may also promote muscle growth or preservation, contributing to overall improvements in body composition [44,45]. These results have implications for the design of exercise programs aimed at optimizing body composition and may inform strategies for weight management and fat loss [44,45]. However, it is important to note that the exact mechanisms underlying these differences require further investigation, and factors such as exercise intensity, duration, and individual characteristics may influence the outcomes. Further research is warranted to elucidate the underlying mechanisms and long-term effects of hypoxic exercise on body composition and overall health.

The beneficial changes in BP of older women may involve reduced risk of hypertension, reduced elasticity of blood vessels, and fluctuations due to hormonal changes, potentially leading to cardiovascular complications [31,46]. The present study investigated the effects of normoxic and hypoxic exercise on BP and arterial stiffness in participants. The results indicated that both normoxic and hypoxic exercise interventions led to favorable changes in BP and arterial stiffness. Specifically, both exercise groups exhibited reductions in SBP, DBP, MAP, PP, and baPWV. These findings are consistent with previous research demonstrating the beneficial effects of exercise on cardiovascular health [47,48,49]. Exercise is known to enhance vascular function, improve endothelial function, and promote vasodilation, leading to reductions in BP and arterial stiffness [50,51]. Several mechanisms may underlie the observed improvements in BP and arterial stiffness following exercise in normoxic and hypoxic conditions. Enhanced nitric oxide bioavailability, increased production of vasodilatory factors, and improved vascular endothelial function are among the key mechanisms implicated in exercise-induced reductions in BP and arterial stiffness [50,51,52,53,54]. Additionally, exercise-induced adaptations in cardiac output, peripheral resistance, and arterial compliance may contribute to the observed improvements in cardiovascular parameters [55,56]. Notably, the HPX group demonstrated greater BP and arterial stiffness reductions than the NMX group. These findings suggest that exercising under hypoxic conditions may confer additional cardiovascular benefits beyond those achieved through normoxic exercise alone. The greater improvements observed in the HPX group may be attributed to the physiological responses elicited by exposure to hypoxia [5,32]. Hypoxic conditions induce adaptations such as increased oxygen delivery, enhanced vasodilation, and improved peripheral circulation, which may contribute to more pronounced reductions in BP and arterial stiffness compared to normoxic exercise [14,57]. Additionally, hypoxic exercise may stimulate greater activation of cardiovascular regulatory mechanisms, leading to more robust improvements in vascular function [14,17,19,35]. These findings highlight the potential of hypoxic exercise as a therapeutic intervention for enhancing cardiovascular health [5]. Further research is needed to elucidate the underlying mechanisms and long-term effects of hypoxic exercise on cardiovascular outcomes.

This study investigated the effects of exercise under normoxic and hypoxic conditions on various biomarkers of inflammation and oxygen transport, including TNF-α, IL-6, EPO, and VEGF. The detrimental changes in TNF-α and IL-6 levels in older women typically involve increased inflammation, while alterations in EPO and VEGF levels may contribute to decreased erythropoiesis and impaired angiogenesis, respectively, potentially impacting overall health and well-being [58,59]. The findings suggest that both normoxic and hypoxic exercise interventions have beneficial effects on biomarkers of inflammation and oxygen transport, albeit with some differences. TNF-α is a pro-inflammatory cytokine involved in immune response and inflammation regulation [60]. Although not showing significant group-by-time interaction effects, TNF-α exhibited significant time effects, indicating changes throughout the intervention. The paired *t*-test results revealed a significant decrease in TNF-α levels in the HPX group, but not in the NMX group. While the difference between groups was not statistically significant, the decrease in TNF-α levels following hypoxic exercise is noteworthy, given the pro-inflammatory role of TNF-α and its association with various cardiometabolic disorders [61,62]. The observed reductions in TNF-α, a marker of inflammation, specifically in the HPX group, further support the notion that exercising in hypoxic conditions may have additional anti-inflammatory benefits compared to normoxic conditions [61,62]. IL-6 is a pro-inflammatory cytokine involved in various physiological processes, including immune response and inflammation regulation [63]. This study found a significant interaction effect for IL-6, indicating that its response to exercise differs between normoxic and hypoxic conditions. Both exercise groups exhibited significant decreases in IL-6 levels over time, suggesting that exercise, regardless of oxygen levels, can attenuate inflammation. However, the magnitude of reduction was more pronounced in the HPX group. This finding aligns with previous research suggesting that hypoxia exposure during exercise may augment anti-inflammatory responses, potentially through mechanisms such as increased production of anti-inflammatory cytokines or enhanced oxygen utilization efficiency [14,28,61]. EPO is a glycoprotein hormone that regulates RBC production and oxygen delivery [64]. Similarly to IL-6, EPO demonstrated significant group-by-time interaction effects, indicating differential responses to exercise conditions. HPX groups exhibited significant increases in EPO levels post-intervention. This finding suggests that exercising in hypoxic conditions may stimulate EPO production to a greater extent than normoxic exercise [65,66,67]. The observed increase in EPO levels in response to exercise aligns with its role in erythropoiesis and oxygen transport, supporting the physiological adaptation to exercise-induced hypoxia [65,66,67,68]. This finding aligns with the physiological response to hypoxia, where the body increases EPO production to stimulate RBC production, thereby improving oxygen delivery to tissues [68,69]. VEGF plays a crucial role in angiogenesis and vasculogenesis, promoting the formation of new blood vessels in response to hypoxic stimuli [70]. VEGF demonstrated significant group-by-time interaction effects, indicating differential responses between the NMX and HPX groups. HPX groups showed significant increases in VEGF levels post-intervention. The greater increase in VEGF levels following hypoxic exercise suggests an enhanced angiogenic response, which may contribute to improved tissue oxygenation and exercise performance [14,32,71]. This suggests that exercising in hypoxic conditions might enhance vascular adaptation and function, potentially improving cardiovascular health [14,32,71]. This study highlights the differential effects of exercise under normoxic and hypoxic conditions on biomarkers of inflammation and oxygen transport, particularly IL-6, EPO, VEGF, and TNF-α. While both exercise modalities positively affected these biomarkers, hypoxic exercise induced more pronounced changes, suggesting its potential superiority in eliciting favorable cardiometabolic adaptations. These findings contribute to our understanding of the physiological responses to exercise in different environmental conditions and have implications for optimizing exercise prescriptions for cardiometabolic health promotion and disease prevention.

The detrimental changes in RBC hemorheological function of older women may entail reduced deformability and aggregation tendency, potentially impairing oxygen delivery and microcirculation [72,73]. The hemorheological properties of RBCs play a crucial role in the delivery of oxygen to tissues during exercise [74]. Understanding how these properties change in response to different environmental conditions, such as normoxia and hypoxia, is important for optimizing exercise performance and assessing physiological adaptations [74,75]. The significant group-by-time interaction effect for RBC EI suggests that the changes in RBC deformability were different between normoxic and hypoxic exercise conditions. This finding aligns with previous research indicating that hypoxia exposure can modulate RBC deformability due to physiological adaptations to low oxygen availability [76,77]. The beneficial changes in RBC EI observed in both exercise conditions suggest improved RBC deformability, which could enhance oxygen delivery to tissues and potentially improve exercise performance [74,75,76,77]. Furthermore, the significant time effects for RBC EI and RBC AI indicate that exercise, regardless of oxygen condition, substantially impacted RBC hemorheological properties. Exercise-induced alterations in blood flow dynamics and shear stress influence RBC deformability and aggregation [8,34]. The observed improvements in RBC EI and reduction in RBC AI reflect the adaptability of RBCs to physiological stressors imposed by exercise, facilitating optimal blood flow and oxygen transport [8,34,74,75,76,77]. Interestingly, there was no statistically significant difference in the group effect, implying that both normoxic and hypoxic exercises elicited comparable changes in RBC hemorheology. The observed improvements in RBC deformability and aggregation suggest that both normoxic and hypoxic exercises can positively influence blood rheology, potentially enhancing exercise performance and cardiovascular health.

Indeed, exploring exercise tolerance in hypoxic conditions and its feasibility, along with considerations of public health access, could provide valuable insights for intervention implementation. By understanding how individuals respond to exercise under low oxygen levels, particularly in high-altitude regions or in scenarios like altitude training or mountain climbing, health-care practitioners can tailor interventions to improve cardiovascular fitness and acclimatization. Moreover, assessing the accessibility of such interventions for broader public health initiatives can inform strategies to promote physical activity in diverse settings and populations, potentially enhancing overall health outcomes in hypoxic environments.

## 5. Conclusions

Our study revealed that 12-week combined exercise training in normobaric hypoxia reduced BMI, percentage body fat, BP, baPWV, TNF-α, and IL-6 and improved FFM, EPO, VEGF, and RBC hemorheological parameters in obese older women. Combined exercise training in normobaric hypoxia could help improve body composition, BP, arterial stiffness, biomarkers of inflammation and oxygen transport, and RBC hemorheological function in obese older women, ultimately leading to better health and cardiovascular function. By exploring these multifaceted outcomes, this research sought to contribute valuable insights into optimizing exercise strategies for managing obesity-related cardiovascular risk factors in aging populations. The findings from this study may hold implications for the development of targeted interventions aimed at improving the overall cardiovascular health of obese older women, thereby addressing a critical health concern in an increasingly aging and overweight global population.

## Figures and Tables

**Table 1:** Physical characteristics of participants.

Variables	NMX	HPX	*p* Value
Age (years)	68.08 ± 0.90	67.83 ± 1.03	0.533
Height (cm)	161.76 ± 5.78	163.23 ± 5.92	0.546
Weight (kg)	70.41 ± 5.30	72.86 ± 5.74	0.289
BMI (kg·m^−2^)	26.87 ± 0.38	27.31 ± 0.66	0.061
Percentage body fat (%)	32.35 ± 1.91	33.23 ± 2.07	0.289

Note. Values are expressed as means ± standard deviations. NMX = normoxia, HPX = hypoxia, BMI = body mass index.

**Table 2:** Combined exercise intervention program.

Program	Contents	Phase (Weeks)	Rep	Duration (min)	Intensity
Warm-up	Dynamic stretching (upper and lower body)	1–12	-	5	
Resistance exercise	Squats, incline chest presses, seated row, push presses, split squats, and pull-aparts (rest for 90 s per set)	1–4	10 (3 set)	Approximately 35	OMNI Resistance Exercise Scale of Perceived Exertion: 7–8
		5–8	12 (3 set)		
		9–12	15 (3 set)		
Rest	-	1–12	-	15	
Aerobic exercise	Treadmill and bicycle (30 min for each exercise)	1–12	-	60	HR_max_: 60–70%
Cool-down	Static stretching (upper and lower body)	1–12	-	5	

Note. HR_max_ = maximal heart rate.

**Table 3:** Changes in body composition between pre- and post-tests in obese older women.

Variables	Group	Pre	Post	Mean Change (95% CI)	F-Value (η_p_^2^)		
					Group	Time	Interaction
Weight (kg)	NMX	70.41 ± 5.30	68.53 ± 4.76	−1.88 *** (−2.68–−1.08)	0.284 (0.013)	86.760 ††† (0.798)	14.798 ††† (0.402)
	HPX	72.86 ± 5.74	68.33 ± 5.11	−4.53 *** (−5.82–−3.24)			
BMI (kg·m^−2^)	NMX	26.87 ± 0.38	26.17 ± 0.49	−0.71 *** (−0.99–−0.43)	0.054 (0.002)	103.405 ††† (0.825)	17.229 ††† (0.439)
	HPX	27.31 ± 0.66	25.62 ± 0.89	−1.68 *** (−2.12–−1.25)			
Fat-free mass (kg)	NMX	45.06 ± 3.39	45.23 ± 3.14	0.17 (−0.33–0.66)	2.243 (0.093)	9.783 †† (0.308)	5.617 † (0.203)
	HPX	46.63 ± 3.67	47.83 ± 3.57	1.20 ** (0.38–2.02)			
Percentage body fat (%)	NMX	32.35 ± 1.91	30.80 ± 1.62	−1.55 *** (−1.86–−1.24)	0.408 (0.018)	431.735 ††† (0.952)	93.026 ††† (0.809)
	HPX	33.23 ± 2.07	29.00 ± 1.53 #	−4.23 *** (−4.76–−3.70)			

Note. Values are expressed as means ± standard deviations. CI = confidence interval, NMX = normoxia, HPX = hypoxia, BMI = body mass index. Significant interaction or main effect: † *p* < 0.05, †† *p* < 0.01, ††† *p* < 0.001. Significant difference between pre- and post-test: ** *p* < 0.01, *** *p* < 0.001. Significant difference between NMX and HPX groups: # *p* < 0.05.

**Table 4:** Changes in blood pressure between pre- and post-tests in obese older women.

Variables	Group	Pre	Post	Mean Change(95% CI)	F-Value (η_p_^2^)		
					Group	Time	Interaction
SBP (mmHg)	NMX	137.68 ± 3.31	135.10 ± 2.79	−2.58 *** (−3.29–−1.88)	4.211 (0.161)	323.418 ††† (0.936)	44.410 ††† (0.669)
	HPX	136.33 ± 4.04	130.70 ± 3.67 ##	−5.63 *** (−6.34–−4.91)			
DBP (mmHg)	NMX	92.91 ± 3.46	92.72 ± 3.54	−0.19 (−0.73–0.35)	2.467 (0.101)	223.983 ††† (0.911)	191.061 ††† (0.897)
	HPX	93.07 ± 3.46	88.24 ± 3.08 ##	−4.83 *** (−5.33–−4.32)			
MAP (mmHg)	NMX	107.83 ± 2.66	106.84 ± 2.50	−0.99 *** (−1.38–−0.60)	5.967 † (0.213)	598.183 ††† (0.965)	272.335 ††† (0.925)
	HPX	107.49 ± 2.44	102.39 ± 2.06 ###	−5.09 *** (−5.48–−4.70)			
PP (mmHg)	NMX	44.78 ± 4.52	42.38 ± 4.60	−2.39 *** (−3.36–−1.42)	0.121 (0.005)	27.425 ††† (0.555)	6.821 † (0.237)
	HPX	43.26 ± 5.80	42.46 ± 5.45	−0.80 (−1.72–0.12)			

Note. Values are expressed as means ± standard deviations. CI = confidence interval, NMX = normoxia, HPX = hypoxia, SBP = systolic blood pressure, DBP = diastolic blood pressure, MAP = mean arterial pressure, PP = pulse pressure. Significant interaction or main effect: † *p* < 0.05, ††† *p* < 0.001. Significant difference between pre- and post-test: *** *p* < 0.001. Significant difference between NMX and HPX groups: ## *p* < 0.01, ### *p* < 0.001.

## Data Availability

The data presented in this study are available upon request from the first or corresponding author.
